# Do We Have Sufficient Evidence to Derive Innovative Approaches to Assessing Unmet Need, Delivering Education on Bladder and Bowel Continence Health, and Providing a Better Environment for Joint Decision‐Making? ICI‐RS 2024

**DOI:** 10.1002/nau.25654

**Published:** 2025-01-13

**Authors:** Nikki Cotterill, Michael Samarinas, Angie Rantell, Caroline Selai, Salvador Arlandis, Kathryn Jones, Paul Abrams, D. Robinson, Adrian Wagg

**Affiliations:** ^1^ School of Health and Social Wellbeing University of the West of England Bristol UK; ^2^ Bristol Urological Institute, North Bristol NHS Trust Bristol UK; ^3^ Urology and Urodynamics Unit, University General Hospital of Larissa Mezourlo Larissa Greece; ^4^ Department of Urogynaecology King's College Hospital NHS Foundation Trust London UK; ^5^ College of Health, Medicine and Life Sciences Brunel University London London UK; ^6^ Department of Uro‐Neurology, Queen Square Institute of Neurology & Psychologist, The National Hospital for Neurology and Neurosurgery/UCLH NHS Foundation Trust University College London London UK; ^7^ Department of Urology, La Fe University and Polytechnic Hospital Valencia University Valencia Spain; ^8^ Department of Medicine, College of Health Sciences, Faculty of Medicine & Dentistry Division of Geriatric Medicine Edmonton Alberta Canada

**Keywords:** continence education, continence promotion, incontinence, shared decision‐making, unmet need

## Abstract

**Context:**

Improved continence outcomes are reliant on identification of unmet need, education delivery, and shared decision‐making. The evidence base on which to derive innovative approaches in these areas was unclear.

**Methods:**

A debate held at the International Consultation on Incontinence‐Research Society meeting, held in Bristol in June 2024, considered ways to improve research requirements to advance these areas.

**Results and Conclusion:**

Artificial intelligence solutions and digital approaches to healthcare are emerging at pace and offer possibilities to improve these three key areas but this must be driven by person‐centered approaches. Care must be taken to avoid increasing inequality through digital exclusion and language barriers. Research questions are highlighted to derive innovation in these three key areas.

## 1 Unmet Needs in Bladder and Bowel Continence Health

Unmet needs in bladder and bowel continence health present significant challenges within healthcare systems. Addressing these needs requires innovative strategies to enhance patient outcomes and optimize healthcare delivery. These unmet needs include economic constraints, patient dissatisfaction, accessibility issues, and the necessity for effective transitional care. From the patient's perspective, reluctance to seek care due to provider related factors and perceived lack of importance of the problem, in addition to practical considerations such as financial and logistical challenges, for example, distance from treatment centers and limited appointment availability, significantly impede patients' ability to access necessary treatments [1]. Additionally, a human‐centered approach is essential to foster confidence between patients and healthcare providers, which is vital for achieving value‐based care and ensuring patient satisfaction [2].

Several factors influence the accessibility and follow‐up care for patients with incontinence. These factors include the patient's age, level of education, disease severity, the competency of the treating healthcare professional, and the underlying disease [3]. Social stigma also deters patients from seeking timely care. Understanding these factors is crucial for developing targeted interventions that improve patient outcomes and satisfaction [4, 5].

Telemedicine and digital technologies have emerged as promising solutions to improve access to and in the delivery of continence care [6]. Online appointments and AI‐driven methods can mitigate logistical challenges, ensuring that patients receive timely and appropriate care [7, 8]. However, caution must be noted and considered during solution development regarding the potential for missed diagnoses in the absence of physical examination or assessment. Revising appointment protocols and adjusting visiting times can enhance accessibility, particularly where medical services may be limited.

Effective transitional care is critical for patients with incontinence, especially those requiring lifelong care. Transitional care ensures continuity and coordination as patients move between different healthcare settings or levels of care [9]. Barriers to successful transition are multifactorial, including patient, provider, and system factors. Addressing these barriers requires a comprehensive approach that includes patient education, improving access to specialized knowledge, and strengthening patient‐therapist relationships [10].

Patients expect healthcare systems to be safe, reliable, accessible, and flexible. To meet these expectations, healthcare providers must reassess clinic workflows to identify and address unmet needs during routine check‐ups [11]. Employing mobile health applications can support the monitoring and management of incontinence, offering patients and caregivers better tools for managing their health [12, 13]. Patient feedback has highlighted the challenges in accessing care for chronic diseases such as bladder and bowel health. Delays in treatment can lead to irreversible disease progression and increase the cost of care for both patients and healthcare systems. By understanding these challenges and implementing innovative strategies, healthcare professionals can mitigate negative outcomes and improve patient access to care [14].

To address the unmet needs in bladder and bowel continence, healthcare providers should consider the following strategies:

− Telemedicine and Online Appointments: Implementing digital platforms for consultations and follow‐ups to improve accessibility.
− Enhanced Education and Patient Information: Providing comprehensive education and available treatments to empower patients. Recent advances are evidenced through the development of apps and continence support websites.
− Strengthening Patient‐Provider Relationships: Fostering trust and communication between patients and healthcare providers.
− Utilizing Digital Technologies: Developing and integrating mobile health applications for continuous monitoring and management, while acknowledging variable levels of health and digital literacy in the population.
− Revising Appointment Protocols: Adjusting clinic schedules and protocols to accommodate more patients and reduce waiting times.

Addressing unmet needs in bladder and bowel continence requires a multifaceted approach that includes enhancing accessibility, leveraging digital technologies, and improving transitional care. Focusing on patient‐centered care and developing innovative solutions will help healthcare systems better meet the needs of patients and improve overall health outcomes. Future research should continue to explore these strategies and their effectiveness in different healthcare settings.

## 2 Education for Bladder and Bowel Continence Health

One of the biggest challenges with regard to education for bladder and bowel continence health is related to the wide variety of populations that require information. There is a need to provide education to the general public to promote health and good bladder and bowel habits, for primary prevention of bladder and bowel conditions but also to encourage help seeking in those with existing problems. For those patients that have been assessed and offered treatment, education regarding specific conditions and their management is required and additional education is often also required for those patients' families and caregivers both professional and nonprofessional. There is also a need for appropriate education for all healthcare professionals with more in‐depth needs for those specializing in bladder and bowel health. Amongst the pediatric populations, there are additional educational needs amongst schools, teachers, and parents. There is also a fundamental need to educate policy makers with regard to the importance of bladder and bowel health to improve services. The Committee which focussed on primary prevention and education at the International Consultation on Incontinence held in 2023 detailed these issues in depth and identified the need for an increased research focus on prevention and education for all including multidisciplinary healthcare professionals, patients, caregivers, and the general public [15].

To derive innovative approaches to education we need to understand what motivates people to learn. For those specialists in the area it is often because of an interest, for some it is due to curiosity or the desire for a challenge, alternatively it may be because of need or necessity or because they are mandated to do so. Case‐based learning, evidence‐based medicine, problem‐based learning, simulation‐based learning, e‐learning, peer‐assisted learning, observational learning, flipped classroom, and team‐based learning are some of the modern learning methodologies in health care [16] but there are no pedagogically validated methods for educational models in continence care.

There have been many different innovations within education, particularly with regard to the role of digital technology. With increasing access to smartphones, apps, and social media, there is increased access to information, for example, the CONfidence app—a self‐help resource for bladder and bowel leakage [17]. There has also been innovation in the role of simulation training and hands‐on models within the healthcare setting. A more recent innovation is in the role of AI in education such as in creating virtual patients for hands‐on training but also within generating content for education and examination questions for objective assessments. However, as with any new technology, there is a need for ongoing research and regulation [18].

To further develop public education, we need to think about what people can and wish to engage with and why. We need to consider how awareness and accessibility of information can be improved at a local, national, and international level. We also need to consider the role of customized/personalized education and resources to meet all health literacy abilities. Further research is required to understand how to improve awareness of new methods of communication and technology to promote self‐management and help‐seeking behaviors. In particular, perceptions regarding incontinence and possibilities for treatment are critical to health‐seeking behaviors [19, 20].

Ultimately, there is still a need for research on both educational content and methodology across all disciplines and an understanding of the evaluation of educational efficacy. We also need further research to improve efficacy of public education acknowledging that multicomponent approaches are required as educational content alone is rarely sufficient [21]. Approaches such as group training, mass marketing, and other techniques also lack an evidence base.

## 3 Shared Decision‐Making (SDM) in Continence Health

**Figure 1:**
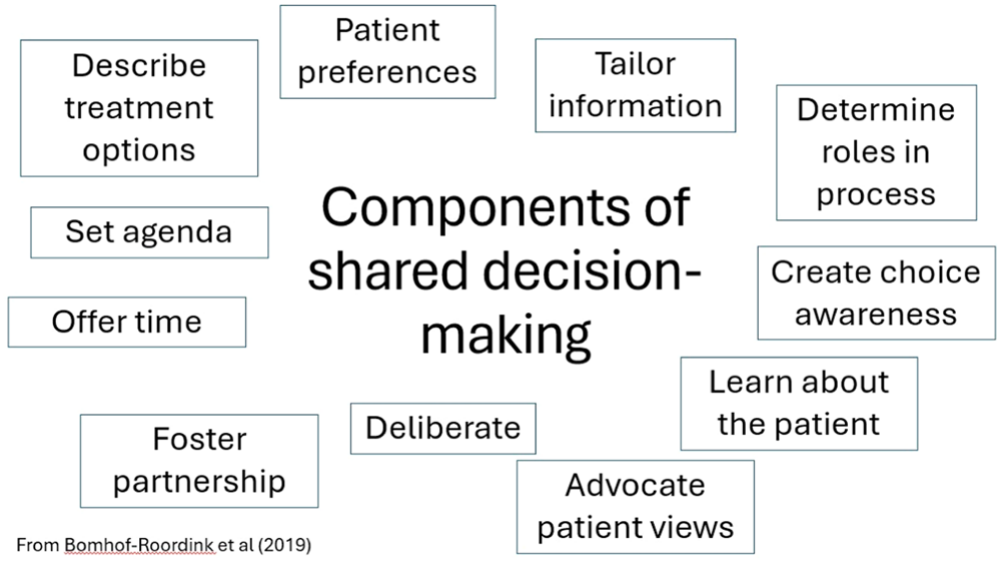
Examples of key components of shared decision‐making.

SDM is an approach where clinicians and patients make decisions together using the best available evidence. Patients are encouraged to think about the available screening, treatment, or management options and the likely benefits and harms of each so that they can communicate their preferences and help select the best course of action for them [22]. To judge whether the benefits and risks of treatment are balanced from a patient's perspective, and to avoid procedures patients would rather not have if they were well informed (and which thus may harm them), clinicians must determine their patients' preferences [23]. A review of the models of SDM, showed that many had key components in common [24]. A selection of the key components are presented in Figure 1.

An American Urological Association (AUA) White Paper on the implementation of SDM in urological practice [25] concludes that SDM represents the state of the art; that patients who have engaged in SDM have greater knowledge, satisfaction, engagement with care, and SDM is an important component of high‐quality healthcare delivery. The benefits of SDM also extend beyond the single therapeutic engagement, as people are more likely to engage in SDM with other clinicians [26]. Therefore, in most circumstances, urologists should adopt SDM into routine clinical practice.

Suitability of SDM in context must also be considered. A German study of SDM in 469 urological patients reported 73% felt highly involved [27]. A more in‐depth qualitative study of post‐partum women with SUI receiving pelvic floor physiotherapy found that some participants preferred SDM with their healthcare provider and others preferred their midwives to make decisions, highlighting the importance of individual preference [28]. This was further explored in an orthopedic population of 115 of patients where 92% preferred a semi‐passive role and desired most involvement in scheduling of surgical treatments [29]. There are so many motivational and contextual factors with different patient groups that affect the patient's decision‐making processes, and therefore, how, or the appetite to, engage with SDM cannot be overlooked. From a clinicians' perspective, the results of a survey of Neurogenic Lower Urinary Tract Dysfunction (NLUTD) care providers from 11 countries were that NLUTD provider practice patterns can widely vary depending upon individual provider's experience and opinions. The researchers found discordances between guideline recommendations, provider practice patterns, and patient‐reported outcome measures. This indicated the need for a decision aid to improve patient‐provider communication and SDM in NLUTD management. Participants said they would use one if available [30].

Two brief tools have been developed to measure SDM in clinical encounters. The items in the CollaboRATE tool are (1) How much effort was made to help you understand your health issues? (2) How much effort was made to listen to the things that matter most to you about your health issues? (3) How much effort was made to include what matters most to you in choosing what to do next? [31]. The second tool is a 9‐item shared decision‐making questionnaire (SDM‐Q‐9) to evaluate the level of involvement in decision‐making, balance of information between treatment options provided, and outcome of the SDM process, and has been translated and validated in Spanish [32].

Key influences on patient/clinician encounters are highlighted that affect the SDM process, which must be recognized in any developments in this area. Discrepancies between recall (40%) and misunderstanding of information provided (48%) highlight the need for effective communication strategies to improve information retention and accuracy [33]. Framing strategies involve presenting information in a way that aligns with patients' cognitive processes, making it easier for them to understand and remember [34]. Decision‐making in functional conditions is often influenced by patients' fear of complications [35, 36]. It is essential to align patient and doctor perspectives to ensure that treatment decisions are made collaboratively and are well‐informed. These factors are also relevant to the education considerations above and highlight the intrinsic link between education, SDM, and subsequently, unmet needs.

Structural barriers can prevent the implementation of SDM, with reportedly at least 10 prerequisites before SDM can become the norm in clinical practice (Table 1).

**Table 1:** Structural requirements for embedded SDM [37].

A favorable policy climate.	Clinical champions
Appropriate regulatory, professional, and legal standards	Evidence of clinical and cost effectiveness
Availability of decision support, including information and tools	Metrics for monitoring progress
Training for clinicians	Financial and other incentives
Patient champions	A feasible implementation plan

Abbreviation: SDM, shared decision making.

Additional system barriers include time, costs and low availability of decision aids. Provider barriers include the challenges posed by presenting multiple, often vastly different, treatment options to patients and the clinicians' loss of personal autonomy [38].

Whilst clinicians are largely in support of embedding SDM in practice [39], there are still challenges to overcome. Decision aids have been shown to increase patient knowledge and decrease decisional conflict by encouraging patients to consider decisions in the context of their values and preferences [40]. By comparison with many other fields of medicine, there have been mixed results in urology. Challenges include ensuring that support materials are comprehensive and provide the relevant information while also representing the best available up‐to‐date evidence [41]. Decision‐making around elective surgery must also endeavor to link medical information with individual experiences and personal criteria, which often change in priority over time [42]. Information alone does not ensure that the patient can competently participate in the decision‐making, especially if the patient is not sure of their role in the interaction, lacks confidence in their communicative abilities, and/or is interacting with a domineering clinician. Some patients have reported that bringing information they have gained from the internet was met with hostility [43]. Methods of information provision also influence encounters with printed information reportedly not empowering patients in the same way as digital resources [44]. The intended audience is critical with most decision aids developed for younger older people (70 years or under) and not specifically tailored for the needs of people with multimorbidity [45].

Healthcare decisions can be among the most complex decisions that individuals face, with an often bewildering array of options and potential outcomes. Research is needed into SDM tools, such as decision aids and ways to eliminate barriers for all parties involved.

## 4 Research Questions to Underpin Innovation

Discussion at the ICI‐RS meeting enabled the identification of key research questions to derive a robust evidence base to underpin innovation as detailed in Table 2.

**Table 2:** Suggested research recommendations to underpin innovation.

**Overarching research questions?**
Can mobile/digital/AI technology assist these innovation intentions? What policy changes/regulatory barriers will facilitate the adoption of innovations in continence care, in particular technological/digital/AI solutions?
**Unmet needs in continence health**
Qualitative and documentary analysis to investigate how healthcare staff can optimize clinic workflows to better identify and address unmet needs in routine bladder and bowel check‐ups? Evidence review and qualitative inquiry to investigate how healthcare staff can contribute to the development and utilization of mobile health applications to monitor and manage continence symptoms? Evidence review to identify strategies healthcare staff can employ to better support the caregivers of patients with incontinence and address their unmet needs? Survey and qualitative study to evaluate the unmet needs in the follow‐up care of pediatric patients with incontinence transitioning to adult care, and how can continuity of care be ensured?
**Education in continence health**
Review underpinning policy to inform how awareness and accessibility to information for all ages and for different cultural groups can be improved at a local/national/international level? An evaluation of cognitive aid strategies for improving patient recall to investigate if identified strategies of information delivery work with larger patient numbers in real‐life settings? Evidence review of the role of customized education/resources particularly considering differing levels of health literacy? Trial to compare new methods of communication for awareness raising to evaluate how effective they are and if impact differs for varying client groups? Public health education to improve awareness of new methods of communication to promote appropriate self‐management and help‐seeking behaviors for UI and FI? Evidence review of the role of technology in public education for continence promotion?
**Shared decision‐making in continence health**
Trial improved communication training to enable healthcare professionals to provide good SDM including framing strategies and ensuring healthcare providers understand the information they provide? In‐depth qualitative investigation of the influence of patients' emotional states during SDM consultations, including information recall, accuracy of recall, treatment decisions, and outcomes? Patient and public engagement initiatives to design and develop tools for patients with low health literacy and those with language barriers? Evidence synthesis of systems that are best at implementing SDM and identification of optimal utilization methods for decision aids to help patients? Trial to investigate if patient preparedness for interventions improves patient reported outcomes? Qualitative inquiry of the inclusion of care partner perspectives in SDM whilst avoiding undue influence?

It is clear that overarching questions exist at a system and technological level. It is suggested that investigating specific research questions as highlighted, will inform the evidence base to address the higher‐level questions.

## 5 Discussion

The opportunity to discuss this topic at this international meeting of thought leaders provided important insights regarding key aspects of improving outcomes for people with incontinence. Importantly, the exploration of the existing evidence base in three key areas identified clear gaps to inform future research directions required to underpin innovative solution development.

Through the exploration of unmet need it emerged that enabling accessibility for all aspects of the continence healthcare journey is critical to optimizing all opportunities for symptom improvement. This includes different approaches to ensure patients can engage with the healthcare provision available, considering digital and remote provision, novel approaches to appointment provision and the development of effective relationships with care providers. Technological and AI solutions could play a part in this innovation journey from a healthcare encounter and workforce scheduling perspective.

Education is a pivotal part of any multicomponent intervention to improve continence outcomes, which applies to patients, carers, health professionals, and policy makers. This discussion highlighted not only the breadth of individuals for consideration but their varying requirements, which adds complexity in deriving resources. In addition, the breadth of the clinical area included was highlighted from prevention education, through to health literacy of individual conditions, and on to management and treatment options. Understanding learning behaviors, motivation, and preferences is also necessary as provision of education will not necessarily result in activation of the knowledge. Uptake and application of education is required, which has a robust evidence base to inform innovation. Technological innovation is to some extent already assisting in this space with mobile health apps for individuals and simulation training for professionals. This has significant potential to transform the learning experience which can be used to maximize impact. Innovating continence education for patients, carers, and healthcare professionals could transform the landscape but requires robust investigation to ensure the education approach and content are evidence based.

SDM is identified as a central tenet of high‐quality care, which is inherently reliant on human factors such as relationship building and trust. Barriers to the implementation of SDM were described and effective communication was highlighted as a core requirement to enable SDM. Tools developed to support this were detailed but concerns are highlighted regarding their applicability to all, in particular, considering people with different educational and language requirements.

Technological innovation opportunities may seem less clearly related to SDM due to the human aspects of the clinician‐patient relationship. Novel innovations to support patient engagement and involvement in SDM through appropriate information provision and gauging understanding has to be considered to remove barriers to the SDM process.

Within the three areas explored, overlapping research directions emerged: healthcare staff have a wealth of expertise and knowledge that they can contribute to innovations to improve continence outcomes; understanding the needs of patients requiring healthcare for incontinence is critical to derive solutions that can address these needs; and human factors also require further investigation for novel solutions to be effective. There are clear opportunities to explore novel ways of meeting the needs of patients from a logistical and educational perspective, which will, in turn, underpin their ability to be effectively involved in SDM as outlined in the proposed research questions.

There is a leaning toward innovation being viewed through the technological and digital lens given advances in this space over recent years. Technology does provide an opportunity for solutions that time‐pressured, and capacity‐limited healthcare staff and systems may not be able to consider. In particular, consideration of the development of resources or user‐led service applications which enable increased accessibility to services, and education to inform decision‐making are highlighted. All research going forward, however, must consider who innovations work for and, often more important, who is excluded and the implications for individuals and society. Therefore, a patient‐centric approach is key to innovation development to ensure the solutions developed are appropriate for the needs of the populations who require them. As digital literacy in the population improves, then the applicability of digital solutions is likely to increase in parallel, however, the needs of rural and remote communities still need to be taken into account in the development of any solution.

Therefore, there is an opportunity to highlight the research required to inform novel developments in this space. It is suggested there is sufficient evidence to underpin the importance of addressing unmet need, education, and SDM requirements for individuals with incontinence and the multidisciplinary workforce who care for them. An opportunity exists to derive new evidence regarding the innovative solutions that could address these requirements and enable improved patient care and outcomes.

## Conflicts of Interest

The authors declare no conflicts of interest.

## Data Availability

The authors have nothing to report.
